# Reusing Waste Coffee Grounds as Electrode Materials: Recent Advances and Future Opportunities

**DOI:** 10.1002/gch2.202200093

**Published:** 2022-10-21

**Authors:** Matthew Pagett, Kar Seng Teng, Geraint Sullivan, Wei Zhang

**Affiliations:** ^1^ Department of Chemical Engineering Swansea University Swansea SA1 8EN UK; ^2^ Department of Electronic and Electrical Engineering Swansea University Swansea SA1 8EN UK; ^3^ SPECIFIC Swansea University Swansea SA1 8EN UK

**Keywords:** circular economy, electrochemical, electrodes, food waste, waste coffee grounds

## Abstract

Coffee industry produces more than eight million tons of waste coffee grounds (WCG) annually. These WCG contain caffeine, tannins, and polyphenols and can be of great environmental concern if not properly disposed of. On the other hand, components of WCG are mainly macromolecular cellulose and lignocellulose, which can be utilized as cheap carbon precursors. Accordingly, various forms of carbon materials have been reportedly synthesized from WCG, including activated carbon, mesoporous carbon, carbon nanosheets, carbon nanotubes, graphene sheet fibers (i.e., graphenated carbon nanotubes), and particle‐like graphene. Upcycling of various biomass and/or waste into value‐added functional materials is of growing significance to offer more sustainable solutions and enable circular economy. In this context, this review offers timely insight on the recent advances of WCG derived carbon as value‐added electrode materials. As electrodes, they have shown to possess excellent electrochemical properties and found applications in capacitor/supercapacitor, batteries, electrochemical sensors, owing to their low cost, high electrical conductivity, polarization, and chemical stability. Collectively, these efforts could represent an environmentally friendly and circular economy approach, which could not only help solve the food waste issue, but also generate high performance carbon‐based materials for many electrochemical applications.

## Graphical abstract

Coffee industry produces more than 8 million tons of waste coffee grounds (WCG) annually. In recent decades, upcycling of WCG‐derived carbon into value‐added electrode materials has attracted growing interests owing to their low cost and high electrochemical properties. Collectively, these efforts could offer more sustainable solutions to global food waste issue and enable circular economy.

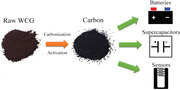

## 1 Introduction

**Figure 1:**
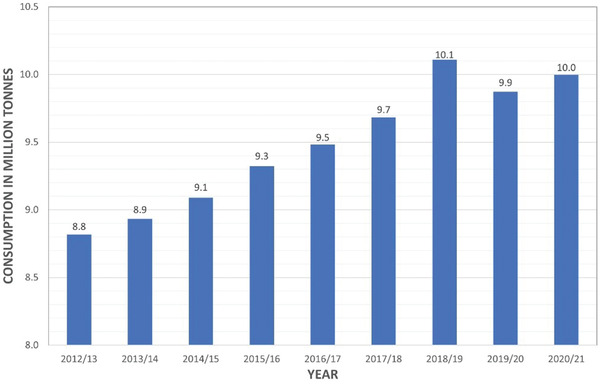
A graph to show the annual consumption of coffee worldwide in million tones.^[^ ^54^ ^]^

Coffee is one of the most popular beverages, with an estimated 2.25 billion cups consumed daily worldwide amounting to over 400 billion cups per year and an annual growth of 1.3% since 2012/2013.^[^ ^50^, ^51^ ^]^ An average cup of coffee has roughly 11 g of ground coffee, which equates to over 9 million tons of ground coffee being brewed worldwide per annum. The global consumption of coffee over the last decade has steadily increased to over 10 million tons by 2021, and this increase is clearly illustrated by **Figure** **1**. For every 1 kg of coffee grounds used within the beverage industry, roughly 0.9 kg is discarded as waste. This has led to millions of tons of waste coffee grounds (WCG) per year.^[^ ^52^ ^]^ When these WCG is disposed of as landfill waste, they decompose and emit methane which is a potent greenhouse gas (25 times more potent than carbon dioxide over 100 years period) with severe adverse effects to the environment.^[^ ^50^ ^]^ In addition, the leachates and soluble from WCG in landfill and via sewage was also found to cause DNA damage and thus induce genotoxicity in aquatic organisms.^[^ ^53^ ^]^

In recent years, the concept of circular economy began to gain traction and most leading countries have been increasing the efforts to shift their economy toward this paradigm. Circular economy aims for economic activities to build and rebuild overall system health by reducing or eliminating the overall amount of waste in society via recycling and reusing products.^[^ ^55^, ^56^ ^]^ As the coffee is mainly disposed as waste after its initial brew and these WCG can pose a significant environmental risk in landfill (i.e., green gas emission and leachate‐induced mutagenicity), it is imperative to find applications of WCG and thus promoting the transition to the circular economy in food industry. On the other hand, as one of the most abundant types of food waste, WCG contains a high volume of valuable sources of carbon and yet remains largely unexploited by industry at the moment. According to a study that investigated chemical, functional, and structural properties of WCG,^[^ ^57^ ^]^ its most abundant components were found to be macro molecules of cellulose and hemicellulose structures which are polymeric structures of hexose saccharides. These two structures can account for 51.50% w/w of the total mass on a dry weight basis. Second highest component was found to be lignin, a highly aromatic structure comprised of crosslinked phenolic structures, which can be up to 40% w/w based on a sulfuric acid hydrolysis extraction.^[^ ^58^ ^]^ The rest of WCG composition includes fatty and amino acids, polyphenols, caffeine, tannins, and minerals,^[^ ^45^ ^]^ etc.

In this context, this review is structured to offer timely insights at the historical development of WCG reuse that occurred throughout the past decades. Mostly notably, WCG is an abundant and cheap carbon resource that can be reused to lower manufacturing costs of electrode materials. Therefore, a more focused view at its most recent applications as high value‐added electrode materials is provided including key milestone publications that marked the start of different application avenues in this promising research area. The key conversion parameters and properties of WCG as electrode materials are then examined, with the important papers being explored and compared in more depth. Finally, some conclusive thoughts and suggestions are outlined to analyze in what directions WCG electrode research may continue and for what other applications it may be used in the future.

## 2 Early Attempts at Recycling WCG

The reuse of WCG has been attempted since the late 1970s in which it was initially reported for using as ruminant feedstock by McNiven et al.^[^ ^59^ ^]^ Sikka et al.^[^ ^60^ ^]^ investigated the use of WCG as a feedstock for fattening pigs. This study showed that an introduction of up to 10% WCG can be included in an isonitrogenous feeding mixture over a period of 70 days without affecting the overall pig's health. In 1996 Udayasekhara^[^ ^61^ ^]^ studied the nutrient composition of WCG and the supplementary effects in animals. It was shown that the increase in spent coffee in the feedstock negatively attributed to the following: gain in body weight, protein efficiency ratio, net protein utilization and amongst others. In addition, WCG has been used in gardening applications as a green compost material, and the composting of the coffee grounds helps to enrich the soil with nitrogen. Provenzano et al. investigated the use of different materials as compost materials, including sawdust, waste coffee, farmyard manure, and the organic fraction of domestic solid waste.^[^ ^62^ ^]^ It was noted that the samples from waste coffee were characterized by a higher functional group heterogeneity and a lower degree of aromaticity. Research in 2004 by Reddy et al.^[^ ^63^ ^]^ demonstrated the organic recycling of WCG through a biotechnological process. This study essentially mixed WCG with biofertilizers and left them to incubate over a period of 45 days. It highlighted that the spent coffee was enriched by the process and therefore it can be reused as organic manure in sustainable agriculture. Alongside agricultural/gardening applications, WCG has also been reported to be used as mulch, a snail/slug deterrent, or as a base for worm food for vermicomposting.^[^ ^64^, ^65^ ^]^

Up to 2000, the reported reuse of WCG in literature was centered in the field of agriculture, as fertilizer and compost. To explore different avenues of WCG reuse, Regalado^[^ ^66^ ^]^ carried out research in the production of enzymes (i.e., β‐mannanases) from solid substrate fermentation of both WCG and copra paste by inoculating both *Aspergillus Oryzae* and *Aspergillus Niger*.^[^ ^66^ ^]^ This was one of the earliest attempts that started to branch out of the agricultural/composting route typically used for coffee waste. Thus far, there has been abundant studies tapping into WCG's potential as feedstock or cultivation medium for the production or extraction of various value‐added microbiological or chemical products,^[^ ^67^ ^]^ including fruiting bodies,^[^ ^68^ ^]^ pigment (e.g., carotenoids^[^ ^69^ ^]^), enzymes,^[^ ^70^, ^71^ ^]^ cosmetics,^[^ ^72^ ^]^ pharmaceutics (e.g., prebiotic,^[^ ^73^ ^]^ anti‐inflammatory^[^ ^74^ ^]^ and antioxidant compounds^[^ ^75^ ^]^), polymers (e.g., polyurethane^[^ ^76^ ^]^ and polyhydroxyalkanoates^[^ ^77^, ^78^ ^]^), and biofuels (e.g., biosyngas,^[^ ^79^ ^]^ biogas,^[^ ^80^ ^]^ biodiesel, and bioethanol^[^ ^81^ ^]^), etc.

In 2001, Nakagawai et al.^[^ ^82^ ^]^ first explored WCG as precursor for activated carbon production via relatively rapid pyrolysis, as previous studies widely used varying food waste materials or natural organic products. In their work, steam activation was used to activate the carbon obtained from WCG, of which surface area 900 m^2^ g^−1^ and pore volume 0.3 cm^3^ g^−1^ were achieved. When samples were further pretreated with a mixture of metal salts and acid treatments (e.g., Ca(OH)_2_ and HNO_3_), these values could be improved to over 1000 m^2^ g^−1^ for surface area and roughly 0.5 cm^3^ g^−1^ for pore volume. Following this research, many studies have demonstrated the use of either raw WCG or WCG‐derived activated carbon as alternative adsorbents for potential environmental remediation, including the removal of heavy metals, dyes, and organic contaminants from aqueous solutions.^[^ ^83^, ^84^, ^85^, ^86^, ^87^, ^88^, ^89^ ^]^ On the other hand, activated carbon is considered to be one of the most used and adaptable electrode materials in electrochemical applications, which has signaled a great potential in the alternative use of WCG in this direction.^[^ ^90^ ^]^ Next, this review continues with a specific focus on how the WCG can be reused in different areas of electrochemical applications, which to our best knowledge has been lacking in the literature.

## 3 Recent Applications of WCG as Electrode Materials

**Figure 2:**
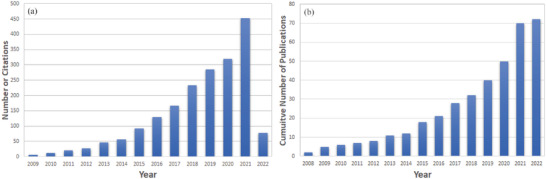
A graph to illustrate a) the number of yearly citations and b) publications of WCG derived carbon materials in the field of electrochemical applications on a cumulative scale to show research interest (last accessed 23/04/2022 on Thomson Reuters’ Web of Science using keywords “waste/spent coffee grounds,” “electrodes” and “electrochemical”).

Literature was searched to gain an insight on the reuse of WCG as high value‐added electrode materials, which yielded 72 publication results from Web of Science between 2008 and 2022. The data collected was then cumulatively plotted to highlight and illustrate years of growth in WCG research for electrochemical applications as shown in **Figure** **2**. It is evident that the interest in the reuse of WCG as electrode materials has steadily grown throughout the years, with a large increase (both citations and publications) occurring recently around 2020–2021 indicating that it is a novel research area and gathering momentum in a variety of applications.

**Figure 3:**
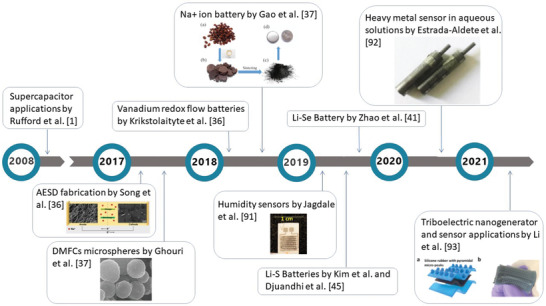
Key milestones in reported studies of recycling WCG as electrode materials. Adapted with permission.^[^ ^34^ ^]^ Copyright 2017, American Chemical Society; Adapted with permission.^[^ ^37^ ^]^ Copyright 2018, Elsevier; Adapted with permission.^[^ ^91^ ^]^ Copyright 2017, Nature; Adapted with permission.^[^ ^92^ ^]^ Copyright 2019, MDPI; Adapted with permission.^[^ ^93^ ^]^ Copyright 2020, Elsevier; Adapted with permission.^[^ ^94^ ^]^ Copyright 2021, Elsevier.

Key milestones in the development of WCG as electrode materials are illustrated in **Figure** **3**. From the application perspective, the reuse of WCG as electrode materials was first reported in 2008 by Rufford et al.^[^ ^1^ ^]^ Nanoporous carbon electrodes were fabricated using pyrolyzed coffee beans with ZnCl_2_ activation and then used as a low‐cost alternative for supercapacitor applications. So far, most of published papers using WCG derived electrodes are in the application of capacitors.

In 2017, reusing pyrolyzed WCG as electrodes began to move away from capacitors: Song et al. attempted to fabricate an asymmetrical energy storage device (AESD) using WCG derived carbon as a cathode for surface‐driven sodium‐ion storage;^[^ ^34^ ^]^ A study by Ghi et al. investigated the use of core (ZnO)/shell (WCG derived carbon) microspheres as a nonprecious electrode material in direct methanol fuel cells (DMFCs).^[^ ^91^ ^]^ In 2018, Krikstolaityte et al. reported reusing of WCG as a promising electrode material for vanadium redox flow batteries, which demonstrated higher energy and voltage efficiency in a static cell test than the typical bipolar graphite (e.g., TF6, SGL Carbon) plates;^[^ ^36^ ^]^ Gao et al. demonstrated WCG derived carbon to be a good candidate as anode material for sodium ion battery.^[^ ^37^ ^]^ In 2019, Zhao et al. fabricated hierarchically microporous activated carbon from WCG enabling Se cathodes for high‐performance lithium–selenium (Li–Se) battery.^[^ ^41^ ^]^ Most recently, Kim et al.^[^ ^40^ ^]^ and Djuandhi et al.^[^ ^45^ ^]^ explored the use of WCG derived biochar as a cheap alternative for a conductive sulfur host in high‐capacity Li/S batteries.

In 2019, Jagdale et al. highlighted a study to use waste coffee biochar after 700 °C pyrolysis as a potential low‐cost carbon precursors for fabricating humidity sensors.^[^ ^92^ ^]^ Screen printing was used to fabricate the sensor electrodes, of which impedance response was observed for relative humidity (RH) between 20% and 100% along with fast response and recovery time (less than 2 min). A study by Estrada‐Aldete et al. in 2020 utilized WCG to build a carbon paste‐based sensor electrode to determine concentrations of heavy metals in aqueous solutions.^[^ ^93^ ^]^ An electrode consisting of 50% WCG provided the best results for differential pulse anodic stripping voltammetry determination of Pb(II) and Cd(II) ions, at limit of detection (LoD) of 90 and 89 × 10^−6^ m, respectively. The chosen electrode was also conducted against a glassy carbon electrode, which showed a significant order of magnitude increase, hence further highlighting the potential use of WCG in electrochemical sensor applications.

**Figure 4:**
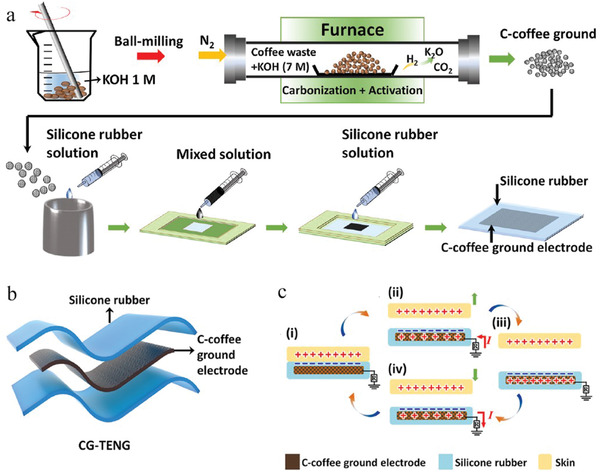
a) Simplified fabrication flowchart for WCG‐derived TENG using processed coffee ground and mixing it with silicone rubber; b) Illustration of the fabricated TENG‐based on WCG‐derived electrode. c) Working mechanism of TENG in the single‐electrode mode. Reproduced with permission.^[^ ^94^ ^]^ Copyright 2021, Elsevier.

In 2021, a paper published by Li et al. outlined, for the first time, the fabrication of a novel, metal‐free and environmentally friendly TENG (Triboelectric Nanogenerator) device by embedding carbonized WCG into the silicone rubber elastomer as a lightweight and shape‐adaptive capacitors (see **Figure** **4**).^[^ ^94^ ^]^ As a result, this wearable TENG device can harvest surrounding energy from human motions and store the generated electricity in these WCG derived capacitors to drive portable electronics. Furthermore, the self‐powered TENG has also demonstrated the capability in sensing human physiological signals, monitoring motions, emulating gestures, as well as developing smart tactile epidermal controller and intelligent vending coaster, energy‐efficient artificial sensors, and wearable electronics toward humanoid robotics and human–machine interfaces.

## 4 Key Parameters of WCG Conversion and its Properties as Electrode Materials

### 4.1 Pyrolytic Carbonization

Within the current literature, WCG derived carbon as electrode materials are noticeably used for electrochemical applications in supercapacitors and batteries. In order to obtain high performance electrodes, WCG need to be carbonized and activated as the former enables its conductivity and the latter will improve the resulting capacitance. Therefore, carbonization and activation processes have been the focus of most reported studies, of which the key conversion parameters and properties across different studies are summarized in **Tables** **1** and **2**. For these applications, WCG was initially pyrolyzed between 500 and 1000 °C, which was commonly carried out in a tube‐furnace. The decomposition temperature of WCG in to activated carbon precursors were reported to be in the range of 257–470  °C.^[^ ^95^ ^]^ More specifically, thermal decomposition of carbohydrates occurs between 200 and 260 °C; that of hemicellulose occurs between 200 and 260 °C; that of cellulose occurs between 240 and 310 °C; that of lignin occurs between 310 and 400 °C.^[^ ^96^, ^97^, ^98^, ^99^, ^100^ ^]^ As the temperature continues to rise (>500 °C), higher degree of graphitization (i.e., lower defect to graphene ratio) will be introduced along with increased electrical conductivity into WCG derived carbon.^[^ ^101^, ^102^, ^103^ ^]^

**Table 1:** Recent works reusing WCG as electrode materials in capacitors

Author	Carbonization	Activation	Surface area	Pore volume	Electrolyte	Capacitance/energy density	Cyclic stability
Rufford et al. 2008^[^ ^1^ ^]^	Dried at 100 °C for 24 h. Carbonized at 900 °C for 1 h under an N_2_ flow	Prior to carbonization, the dried samples were mixed in an equal ration of ZnCl_2_	CGC: 1019 m^2^ g^−1^ Maxsorb: 1840 m^2^ g^−1^	CGC *V*_Total_: 0.48 cm^3^ g^−1^ Maxsorb *V*_Total_: 0.48 cm^3^ g^−1^	1 m H_2_SO_4_	368 F g^−1^ (0.0 gA g^−1^) >10 Wh kg^−1^ (≤6000 W kg^−1^)	Steady performance over 10 000 cycles
Rufford et al. 2009^[^ ^2^ ^]^	Carbonized at 900 °C for 1 h under a N_2_ atmosphere	Prior to carbonization dried coffee grounds were mixed with ZnCl_2_ at ratios of 1.0, 3.5, and 5.0	1.0:1019 m^2^ g^−1^ 3.5:940 m^2^ g^−1^ 5.0:1021 m^2^ g^−1^	5.0 *V*_Total_: 1.30 cm^3^ g^−1^ *V*_mic_: 0.35 cm^3^ g^−1^ *V*_meso_: 0.95 cm^3^ g^−1^	1 m TEABF_4_/AN	1.0: ≈100 F g^−1^ ≈50 F g^−1^ 3.5: ≈130 F g^−1^ ≈110 F g^−1^ 5.0: ≈135 F g^−1^ ≈125 F g^−1^ Each sample was measured at 0.1 and 1 A g^−1^, respectively	There was a large capacitance drop at over 1 A g^−1^
Rufford et al. 2010^[^ ^3^ ^]^	Dried at 100 °C for 24 h Carbonized at 900 °C for 1 h under an N_2_ flow	Dried samples were mixed with an equal mass of FeCl_3_/MgCl_2_/ZnCl_2_ prior to carbonization	0: <10 m^2^ g^−1^ Zn: 977 m^2^ g^−1^ m^2^ g^−1^ Mg: 123 m^2^ g^−1^ m^2^ g^−1^ Fe: 846 m^2^ g^−1^	Fe: 0.64 cm^3^ g^−1^ *V*_mic_: 0.21 cm^3^ g^−1^ *V*_meso_: 0.43 cm^3^ g^−1^	1 m H_2_SO_4_	Zn: ≈82 F g^−1^ ≈55 F g^−1^ Fe: ≈52 F g^−1^ ≈45 F g^−1^ Each sample was measured at 0.1 A g^−1^ and 10 A g^−1^, respectively.	Zn: 93% (5000c.) Fe: 99% (5000c.)
Nickolov et al. 2011^[^ ^4^ ^]^	Pretreated at 573 K in air for 4 h and then carbonized at 943, 983, 1023, 1073, 1223 K for 90 min	Done in one step along with carbonization. Material mixed with KOH in 1:1 and 1:1.2 ratios	1223 K: 689 m^2^ g^−1^ 1073 K: 1126 m^2^ g^−1^ 1023 K: 731 m^2^ g^−1^	1223 K: 0.29 cm^3^ g^−1^ 1073 K: 0.52 cm^3^ g^−1^ 1023 K: 0.33 cm^3^ g^−1^	Et_4_NBF_4_‐PC	1223 K: 17 F g^−1^ 1073 K: 32 F g^−1^ 1023 K: 21 F g^−1^	n.d.
Huang et al. 2013^[^ ^5^ ^]^	No specific methods discussed	H_3_PO_4_ was used to activate the carbons at impregnation ratios 0.5, 0.6, 1.0, 2.0, and 3.0	0.5: 330 m^2^ g^−1^ 1.0: 763 m^2^ g^−1^ 3.0: 554 m^2^ g^−1^	0.5 *V*_Total_ 0.18 cm^3^ g^−1^ 1.0 *V*_Total_ 0.42 cm^3^ g^−1^ 3.0 *V*_Total_ 0.35 cm^3^ g^−1^	1 m H_2_SO_4_	0.5:160 F g^−1^ 142 F g^−1^ 1.0:167 F g^−1^ 145 F g^−1^ 2.0:180 F g^−1^ 157 F g^−1^ Each sample was measured at 0.05 A g^−1^ and 1 A g^−1^ respectively.	82% after 10,000 cycles at 5 A g^−1^ and a potential window of 1.5 V
Kikuchi et al. 2013^[^ ^6^ ^]^	Dried at 105 °C for ≈12 h and then carbonized at 800 °C for 1 h under N_2_ flow	Mixed with 2 m/5 m KOH (30 g/560 mL)	2 m KOH: 1854 m^2^ g^−1^ 5 m KOH: 1825 m^2^ g^−1^	n.d.	1 m TEMA‐BF_4_/PC	2 m KOH: 80 F g^−1^ 55 F g^−1^ 5 m KOH: 100 F g^−1^ 80 F g^−1^ Commercial: 110 F g^−1^ 40 F g^−1^ Each sample was measured at 50 and 150 mA cm^−2^, respectively.	The potassium‐based carbons had higher retention over the commercial carbons.
Tashima et al. 2014^[^ ^7^ ^]^	Dried at 150 °C for 1 h and pyrolyzed in tube furnace under 600 °C for 1 h at either 10 °C min^−1^ or 5 °C min^−1^ with N_2_ flow (700 mL min^−1^)	Reintroduced to tube furnace at 1000 °C, with CO_2_ flow (700 mL min^−1^) for varying times (1–6 h)	1264 m^2^ g^−1^ (10 °C min^−1^, 3 h) 1596 m^2^ g^−1^ (10 °C min^−1^, 2 h) 1867 m^2^ g^−1^ (5 °C min^−1^, 2 h)	*V*_mic_: 0.769 cm^3^ g^−1^ *V*_meso_: 0.305 cm^3^ g^−1^ (10 °C min^−1^, 2 h) *V*_mic_: 0.958 cm^3^ g^−1^ *V*_meso_: 0.561 cm^3^ g^−1^ (5 °C min^−1^, 2 h)	0.8 m [(C_2_H_5_)_4_NBF_4_] In propylene carbonate.	103 F g^−1^ 0.8 m [(C_2_H_5_)_4_NBF_4_] 183 F g^−1^ 0.5 m H_2_SO_4_	Little variance was reported over 3000 cycles.
Wang et al. 2015^[^ ^8^ ^]^	Microwave plasma irradiation (1 torr, 1:1 H_2_/Ar mix, 2.45 GHz 900 W microwave)	No activation step	n.d.	n.d.	1 m KCl	1293.33 µF cm^−2^ ≈20 times higher than glassy carbon electrode	n.d.
Ramasahayam et al. 2015^[^ ^9^ ^]^	Placed in a 2.45 GHz and 1.25 kW microwave reactor for 30 min with estimated temperatures over 1000 ^o^C	Coffee was dried and grounded with different ratios of ammonium phosphate in a mortar and pestle	PNDC‐1: 362.82 m^2^ g^−1^ PNDC‐2: 648.54 m^2^ g^−^ PNDC‐3: 999.64 m^2^ g^−1^	PNDC‐3: *V*_Total_: 0.57 cm^3^ g^−1^ *V*_mic_: 0.14 cm^3^ g^−1^ *V*_meso_: 0.43 cm^3^ g^−1^	1 m H_2_SO_4_	PNDC‐1: 117 F g^−1^ PNDC‐2: 184 F g^−1^ PNDC‐3: 286 F g^−1^ (5 mV s^−1^)	98% across 2000 cycles.
Yang et al. 2015^[^ ^10^ ^]^	Heat treated at 600, 700, and 800 °C for an h under an inert atmosphere	Mixed with KOH (ratio of 2) and activated under Ar atmosphere and 800 °C for 1 h	600: 2558 m^2^ g^−1^ 700: 2280 m^2^ g^−1^ 800: 1527 m^2^ g^−1^	600: 1.01 cm^3^ g^−1^ 700: 0.86 cm^3^ g^−1^ 800: 0.54 cm^3^ g^−1^ (*V*_mic_)	1 m (C_2_H_5_)_4_NBF_4_.	n.d.	All samples exceeded 87%
Yun et al. 2015^[^ ^11^ ^]^	800 °C carbonization for 2 h	Ultrasonicated in *N,N‐*dimethylformamide and then frozen at ‐196 °C; Following freeze drying at ‐45 °C and 4.5 Pa for 72 h, 5 g of WCG was mixed with 2.5, 5 and 10 g KOH in a mortar and pestle	1945.7 m^2^ g^−1^	*V*_mic_: 1.146 cm^3^ g^−1^ *V*_meso_: 0.705 cm^3^ g^−1^	BMIM BF_4_/AN	121 F g^−1^ (0.5 A g^−1^) 34.5 Wh kg^−1^ (11 250 W kg^−1^)	90.5% over 5000 cycles.
An et al. 2015^[^ ^12^ ^]^	Pretreated at 280 °C for 2 h and then carbonized at 800 °C for 2 h under N_2_ gas.	No activation step	CNF: 443 m^2^ g^−1^ PCNF: 1071 m^2^ g^−1^ Pt/CNF: 245 m^2^ g^−1^ Pt/PCNF:670 m^2^ g^−1^	CNF: 0.22 cm^3^ g^−1^ (84.3% micro) PCNF: 0.61 cm^3^ g^−1^ (82.8% micro)	6 m KOH	CNF: 37.5 F g^−1^ PCNF: 118.7 F g^−1^ Pt/CNF: 50.0 F g^−1^ Pt/PCNF: 130.2 F g^−1^	CNF: 80% PCNF: 83% Pt/CNF: 91% Pt/PCNF 93%
Wang et al. 2016^[^ ^13^ ^]^	Dried at 40 °C for 48 h Stirred with 1 m KOH for 6 h and dried further at 50 °C for 24 h	Mixed with an equal weight of KOH, and pyrolyzed at 600, 700, and 800 °C for 2 h under N_2_ atmosphere	600: 663 m^2^ g^−1^ 700: 1622.77 m^2^ g^−1^ 800: 1206.38 m^2^ g^−1^	Micropore *S*_BET_: 890.41 m^2^ g^−1^ (700 °C) Mesopore *S*_BET_: 732.36 m^2^ g^−1^ (700 °C)	6 m KOH	700: 475 F g^−1^ (0.1 A g^−1^) 175 F g^−1^ (1 A g^−1^) 11 Wh kg^−1^ (7500 W kg^−1^)	Excellent stability with little to no decay over 10 000 cycles.
Park et al. 2016^[^ ^14^ ^]^	Dried at 80 °C for 24 h and then pyrolyzed in tube furnace at 800 °C for 2 h under N_2_ flow	Mixed with KOH prior to pyrolysis	1960.1 m^2^ g^−1^	Micropore *S*_BET_: 1932.5 m^2^ g^−1^ Mesopore *S*_BET_: 27.6 m^2^ g^−1^	6 m NaOH (1) 1 m Na_2_SO_4_ (2)	438.5 F g^−1^ (1) 287.9 F g^−1^ (2)	81.9% (1) 75.7% (2) (2000 cycles)
Choi et al. 2018^[^ ^15^ ^]^	Dried at 100 °C for 24 h and then precarbonized at 700 °C for 2 h under Ar atmosphere	Mixed with different ratios of KOH (0.5–6) and the pyrolyzed at 700 °C for 2 h under an Ar atmosphere	≈2614 m^2^ g^−1^	*V*_Total_ : 0.79 cm^3^ g^−1^	5 m KOH or EMIM‐TFSI	125–135 F g^−1^ (0.5 A g^−1^) 187.57 Wh kg^−1^ (438 W kg^−1^ density)	87% retention after 10 000 cycles
Srinu et al. 2018^[^ ^16^ ^]^	Obtained coffee carbon (CC) was precarbonized before use in this work	The CC was doped using melamine and triphenyl phosphate to create N‐CC, P‐CC, N/P‐CC	CC: 279.91 m^2^ g^−1^ N‐CC: 454.52 m^2^ g^−1^ P‐CC: 821.56 m^2^ g^−1^ N/P‐CC: 1018.13 m^2^ g^−1^	CC: 0.108 cm^3^ g^−1^ N‐CC: 0.118 cm^3^ g^−1^ P‐CC: 0.248 cm^3^ g^−1^ N/P‐CC: 0.213 cm^3^ g^−1^	0.1 m KOH	n.d.	Drop of 30 mV over 10,000 cycles compared to Pt/C's 180 mV in 1000 cycles
Choi et al. 2019^[^ ^17^ ^]^	Pyrolysis in tube furnace (800 °C, 5 °C min^−1^, 2 h, N_2_ rich atmosphere)	KOH 1:1 ratio	1824.0 m^2^ g^−1^	0.529 cm^3^ g^−1^ (*V*_Total_)	3 m KOH	148 F g^−1^ (0.5 A g^−1^) Power density of 12.8 kW kg^−1^	97% (10 000 cycles)
Chiu and Lin 2019^[^ ^18^ ^]^	Pyrolysis in tube furnace at 700 °C for 2 h under N_2_	1 g of WCG was dispersed in 5 mL of solution containing 2 g of activating agent (H_3_PO_4_, HCl, FeCl_3_, ZnCl_2_, NaOH and KOH)	H_3_PO_4_ – 75 m^2^ g^−1^ HCl – 9 m^2^ g^−1^ FeCl_3_ – 41 m^2^ g^−1^ ZnCl_2_ – 1242 m^2^ g^−1^ NaOH – 669 m^2^ g^−1^ KOH – 1250 m^2^ g^−1^	KOH – 0.173 cm^3^ g^−1^ ZnCl_2_ – 0.321 cm^3^ g^−1^ NaOH – 0.173 cm^3^ g^−1^ H_3_PO_4_ – 0.5 cm^3^ g^−1^ HCl – 0.5 cm^3^ g^−1^ FeCl_3_ – 0.5 cm^3^ g^−1^	2 m KOH	H_3_PO_4_ – 51.0 F g^−1^ HCl – 0.6 F g^−1^ FeCl_3_ – 41.3 F g^−1^ ZnCl_2_ – 72.9 F g^−1^ NaOH – 69.5 F g^−1^ KOH – 105.3 F g^−1^	Coulombic efficiency of >80% over 8000 repeated charging and discharging
Jung et al. 2020^[^ ^19^ ^]^	Dried at 105 °C for 24 h and treated in hydrothermal batch reactor (80% moisture content) at 230 °C for 4 h	Mixed with 1 m KOH at a ratio of 1:3.5 and then heated to 700 °C for 2 h	7.0164 m^2^ g^−1^ (nonactivated 1067.114 m^2^ g^−1^ (activated)	n.d.	1 m Li_2_SO_4_	Activated: 140 F g^−1^ Nonactivated: 5 F g^−1^ (1 mV s^−1^ scan rate)	97% retention 1500 cycles at 0.3 A g^−1^
Ghi et al. 2020^[^ ^20^ ^]^	Dried at 50 °C for 6 h and then pyrolyzed in tube furnace at 700 °C for 2 h under N_2_ gas flow	Prior to carbonization, 1:1 ratio of ZnCl_2_ was used in 500 mL H_2_O	Nonactivated: 6.1 m^2^ g^−1^ Activated: 212 m^2^ g^−1^	Nonactivated: 0.019 cm^3^ g^−1^ Activated: 1.12 cm^3^ g^−1^	Electrolyte containing ethanol	Current density: 35 mA cm^−2^ at 0.4 V	n.d.
Liu et al. 2020^[^ ^21^ ^]^	Dried under vacuum at 80 °C for 6 h and pyrolyzed in tube furnace at 700 °C for 2 h under Ar gas	0.6 g of FeCl_3_ (per 3.0 g WCG), and varying mass KOH (per 0.3 g WCG) in 30 mL of H_2_O with 30 min sonication and 72 h freezing prior to carbonization	No KOH: 801 m^2^ g^−1^ 4.0 Ratio KOH: 3459 m^2^ g^−1^	No KOH: *V*_Total_: 1.03 cm^3^ g^−1^ 4.0 Ratio KOH: *V*_Total_: 2.41 cm^3^ g^−1^ *V*_mic_: 0.64 cm^3^ g^−1^ *V*_meso_: 1.64 cm^3^ g^−1^	6 m KOH EMIMBF_4_	466 F g^−1^ at 1 mV s^−1^ (6 m KOH)	67% capacitance retention.
Biegun et al. 2020^[^ ^22^ ^]^	Hydrolysis in an autoclave at 180 °C for 12 h followed by primary carbonization at 600 °C for 2 h in inert atmosphere	Mixed with KOH (4:1 KOH/material) and activated at 800 °C for 1 h in an inert atmosphere	2906 ± 19 m^2^ g^−1^	Total pore volume – 1.44 cm^3^ g^−1^ (mainly micropores present within the range of 0.6–0.8 nm)	PYR13‐TFSI	180.1 ± 15.0 F g^−1^ (0.1 A g^−1^) 178 ± 12.0 (A g^−1^)	n.d.
Kikuchi et al. 2021^[^ ^23^ ^]^	Dried at 110 °C and pyrolyzed in electric furnace at 600 °C for 1 h under N_2_ flow	After pyrolysis, 2 g of samples was heated to 850 °C and activated by steam from a saturated steam generator	n.d.	n.d. mesoporous structures resulting from activation	1 m TEMA‐BF_4_	92.3 F g^−1^ at 5 mA cm^−2^ (after 3 h of activation)	n.d.
Hossain et al.2021^[^ ^24^ ^]^	Dried at 120 °C for 3 h, which was followed by carbonized at 600 °C for 20 min under an N_2_ gas flow	CO_2_ was used as an oxidizing agent for 1 h under a 1 L min^−1^ flow rate at 650 and 750 °C, respectively	579.9 ± 8.2 m^2^ g^−1^ (sample activated at 750 °C)	*V*_Total_: 0.365 cm^3^ g^−1^ *V*_mic_: 0.172 cm^3^ g^−1^ *V*_meso_: 0.193 cm^3^ g^−1^ (750 °C)	5 m KOH	Reported up to 190 F g^−1^ (scan rate 5 mV s^−1^)	≈92% (2000 cycles)
Hieh et al. 2021^[^ ^25^ ^]^	Demineralized in HCl at 80 °C for 4 h and then carbonized at 600 °C for 3 h in Ar	1 g of the sample was heated up to 900–950 °C, when CO_2_ was introduced for 3 h	900: 1200 m^2^ g^−1^ 925: 1800 m^2^ g^−1^ 950: 2500 m^2^ g^−1^	900: 0.63 cm^3^ g^−1^ 925: 0.95 cm^3^ g^−1^ 950: 1.28 cm^3^ g^−1^ (*V*_Total_)	1.5 m LiClO_4_ in propylene carbonate.	950 performed best 22.4 F g^−1^ (0.5 A g^−1^) 192.4 F g^−1^ (1.0 A g^−1^) 155.6 F g^−1^ (3.0 A g^−1^)	80.8% over 30 000 cycles.
Poochai et al. 2021^[^ ^26^ ^]^	Dried at 80 °C for 24 h and then pyrolyzed at 700 °C for 1 h under a N_2_ atmosphere	WCG mixed with KOH (1:1)	NPC: 706.5 m^2^ g^−1^ 3% C/N: 1158 m^2^ g^−1^ CNT: 228.9 m^2^ g^−1^	NPC: 0.23 cm^3^ g^−1^ 3% C/N: 0.38 cm^3^ g^−1^ CNT: 1.17 cm^3^ g^−1^	1 m TEABF_4_/ACN	NPC: 74 F g^−1^ 3% C/N: 132 F g^−1^ CNT: 48 F g^−1^	Retention of 85% after 10 000 cycles at 3 A g^−1^
Adan‐Mas et al. 2021^[^ ^27^ ^]^	Pyrolysis in quartz reactor at 700, 750, and 800 °C for 1 h under an N_2_ flow. (Noted PA.)	Mixed with KOH (1:1.5) and placed in a tubular oven at 850 °C for 30 and 60 min. (Noted CA)	PA3: 981.12 m^2^ g^−1^ CA1: 1602.23 m^2^ g^−1^ CA2: 2330.17 m^2^ g^−1^	PA3: 1.03 cm^3^ g^−1^ CA1: 0.67 cm^3^ g^−1^ CA2: 1.17 cm^3^ g^−1^ (*V*_Total_)	1 m Na_2_SO_4_	PA3: 72 F g^−1^ (1.0 Ag^−1^) CA3: 84 F g^−1^ (1.0 Ag^−1^)	PA3: 92% (4000 c. 10 Ag^−1^) CA3: 85% (5000 c. 10 Ag^−1^)
Gissawong et al. 2021^[^ ^28^ ^]^	Dried at 80 °C for 2 h and then treated in hydrothermal autoclave at 150 °C for 30 min	60 mL of 1 m KOH was used in tandem with pyrolysis	n.d.	n.d.	5 × 10^−3^ m ^[^Fe(CN)_6_]^3‐/4−^ Containing 0.1 m KCl	n.d.	RSD, *n =* 5 Intraday 3.8% Interday 8.6%
Lee et al. 2021^[^ ^29^ ^]^	Dried at 80 °C for 24 h and then carbonized at 600 °C under N_2_ flow	Mixed with KOH and placed in tube furnace at 900 °C for 1 h under N_2_ atmosphere	3065.0 m^2^ g^−1^ (8:1 ratio of KOH to WCG)	*V*_Total_: 3.797 cm^3^ g^−1^ *V*_mic_: 1.337 cm^3^ g^−1^ *V*_meso_: 2.461 cm^3^ g^−1^	0.5 m Na_2_SO_4_	127.8 F g^−1^ at 1 A g^−1^ density	82% after 2500 cycles
Konnerth et al. 2021^[^ ^30^ ^]^	Dried at 105 °C overnight and treated in an autoclave at 220 °C for 2 h with 150 g H_2_O/25 g biomass	K_2_CO_3_ (2:1 ratio to waste) under N_2_ flow (20 L min^−1^)	n.d.	n.d.	0.5 m H_2_SO_4_	MnO_2_ doped: 87.0 F g^−1^ Pure AC: 40.3 F g^−1^	n.d.
Dericiler et al. 2022^[^ ^31^ ^]^	Dried at 80 °C overnight and pyrolyzed in rotating furnace at 1000 °C for 5 s	No activation step	n.d.	n.d.	6 m KOH	Recycled graphene oxide: 0.42 F g^−1^, 0.39 F g^−1^	93% retention between 1st and 50th cycles.
Wannasri et al. 2022^[^ ^32^ ^]^	Dried at 80 °C for 2 h and then treated in hydrothermal autoclave at 150 °C for 30 min	No activation step	n.d.	n.d.	5 × 10^−3^ m ^[^Fe(CN)_6_^]^^3−/4−^	n.d.	n.d.
Alhnidi et al. 2022^[^ ^33^ ^]^	Hydrothermal treatment at 220 °C for 5 h, followed by pyrolysis at 600 °C for 2 h under N_2_ flow	Urea and alanine were used as precursors to enrich the carbon with N content	Nitrogen enriched: 51 m^2^ Unaltered samples: 2.3 m^2^	Nitrogen enriched: 0.053 cm^3^ g^−1^ Unaltered samples: 0.008 cm^3^ g^−1^	0.5 m H_2_SO_4_	Nitrogen enriched: 40 F g^−1^ (5 mV s^−1^) Unaltered samples: ≈8 F g^−1^ (5 mV s^−1^)	n.d.

**Table 2:** Recent works reusing WCG as electrode materials in various forms of batteries

Author	Carbonization	Activation	Surface area	Pore size/volume	Electrolyte	Reversible capacity	Coulombic efficiency
Song et al. 2017^[^ ^34^ ^]^	800 °C carbonization for 2 h	Ultrasonicated in *N,N‐*dimethylformamide and then frozen at −196 °C; Following freeze drying at −45 °C and 4.5 Pa for 72 h, 5 g of WCG was mixed with 2.5 g KOH in a mortar and pestle.	Functionalized 1764.8 m^2^ g^−1^ Hierarchically porous 123.2 m^2^ g^−1^	n.d.	1 m NaPF_6_ in an EC/PC mixture (1:1 v/v)	130 mAh g^−1^ (0.1 A g^−1^) (functionalized) 105 mAh g^−1^ (15 A g^−1^) (hierarchically porous)	65% over a 100‐fold current density increase and over 1000 cycles
Zhao et al. 2017^[^ ^35^ ^]^	Dried at 60 °C under vacuum for 12 h and then carbonized at 900 °C for 3 h under Ar flow	Precarbonization mixed with a 1:1 ratio of ZnCl_2_ and ball milled at 200 rpm for 3 h.	Pre sulfur 1017.5 m^2^ g^−1^ post sulfur 22.4 m^2^ g^−1^	Pre sulfur 0.480 cm^3^ g^−1^ post sulfur 0.015 cm^3^ g^−1^	1 m LiCF_3_SO_4_ in dimethoxy ethane and 1,3‐dioxolane	1st 1206 mAh g^−1^ 2nd 1073 mAh g^−1^ Xth 613 mAh g^−1^	57% from 2nd cycle after 100 cycles. Plateau @ 20c.
Krikstolaityte et al. 2018^[^ ^36^ ^]^	Dried at 105 °C overnight and then pyrolyzed in electric furnace (850 °C, 5 °C min^−1^, 0.5 h, N_2_ flow 250 mL min^−1^)	Introduction of steam (63% vol) at 1 h intervals (up to 3 h) to carbonization step	BC: <1 m^2^ g^−1^ AC1: 541 m^2^ g^−1^ AC2: 817 m^2^ g^−1^ AC3: 1113 m^2^ g^−1^	BC: <0.01 cm^3^ g^−1^ AC1: 0.29 cm^3^ g^−1^ AC2: 0.55 cm^3^ g^−1^ AC3: 0.75 cm^3^ g^−1^	1.6 m V^3+/4+^ containing 4 m H_2_SO_4_	n.d.	n.d.
Gao et al. 2018^[^ ^37^ ^]^	Dried at 150 °C for 2 h and then carbonized at 600–900 °C for 1 h under flowing Argon	No activation step	95.53 m^2^ g^−1^	n.d. 100–300 Å pore width	Mainly 1 m NaClO_4_ in TEGDME	154.2 mAh g^−1^ at 200 mA g^−1^ (after 50 cycles)	91.7% over the 50 cycles
Um et al. 2018^[^ ^38^ ^]^	Carbonization at 600 °C for 2 h under a N_2_ gas flow.	Steam was used as an activating agent at a rate of 2 mL g^−1^ of char per h. At 800 °C for 2 h.	503 m^2^ g^−1^	n.d.	1 m LiPF_6_ in EC/DEC	≈200 mAh g^−1^ after 100 cycles (0.1 A g^−1^)	Reported as “excellent stability”
Tsai et al. 2019^[^ ^39^ ^]^	Pretreated at 500 °C for 5 h, which was followed by calcination at 800 °C for 2 h under Ar flow	For N‐enriched carbons, HMT (hexamethylene‐tetramine) were mixed and calcined at 800 °C for 2 h under Ar flow.	N‐CGC: 1.863 m^2^ g^−1^ CGC: 0.5407 m^2^ g^−1^	N‐CGC: 0.0075 cm^3^ g^−1^ CGC: 0.0026 cm^3^ g^−1^	1 m LiPF_6_ in EC/EMC 1 m NaClO_4_ in ED/DEC	CGC: 278 mAh g^−1^ N_10_CGC: 372 mAh g^−1^ N_20_CGC: 397 mAh g^−1^	Over 3 cycles the columbic efficiency was stated as. CGC: 49.1% N_10_CGC: 53.8% N_20_CGC: 58.0%
Kim et al. 2019^[^ ^40^ ^]^	Dried at 110 °C for 24 h and then pyrolyzed in tube furnace at 800 °C for 24 h under an N_2_ flow (250 mL min^−1^)	After carbonization, 2 m HNO_3_ and 3 w/w% H_2_O_2_ (50 mL g^−1^) for 24 h at 60, 80, and 100 ^o^C	Dry0: 31.69 m^2^ g^−1^ Dry80: 256.34 m^2^ g^−1^ Wet80: 1037.52 m^2^ g^−1^	W80 *V*_mic_ – 0.378 cm^3^ g^−1^ D80 *V*_mic_ – 0.239 cm^3^ g^−1^	1 m LITFSI and 0.2 m LiNO_3_ dissolved in DOL and DME	Wet80: 1458 mAh g^−1^ (first cycle) 474 mAh g^−1^ (400 cycles)	Average reduction of 0.3% per cycle (over 400 cycles)
Zhao et al. 2019^[^ ^41^ ^]^	Dried at 80 °C overnight and then pyrolyzed in tube furnace at 700 °C for 2 h under N_2_ atmosphere	Mixed with KOH (2:1 w/w) and annealed at 700, 800, and 900 °C for 2 h under N_2_ atmosphere.	700–1355 m^2^ g^−1^ 800–981 m^2^ g^−1^ 900–978 m^2^ g^−1^	700 °C *V*_meso_ 0.52 cm^3^ g^−1^ 900 °C *V*_meso_ 0.31 cm^3^ g^−1^	1 m LiPF_6_ in EC and DEC	700–655 mAh g^−1^ 800–424 mAh g^−1^ 900–371 mAh g^−1^	All cathodes experienced a sharp drop between 1st and 2nd, but maintained at 99.5%
Fernandez et al. 2019^[^ ^42^ ^]^	Sterilization and flash pyrolysis in furnace at 700 °C	No activation step	PA‐700: 641.27 m^2^ g^−1^	PA‐700: *V*_Total_: 0.329 cm^3^ g^−1^ *V*_mic_: 0.232 cm^3^ g^−1^	Saltwater	60 Wh was calculated as well as a 5–30 V/3 A/10–60 W output	Battery was able to play a radio for roughly 4 h
Kang et al. 2019^[^ ^43^ ^]^	Pyrolysis at 800 °C for 2 h under an Ar flow	Prior to carbonization, coffee waste and NaCrO_2_ powder were mixed in a ball mill at 300 rpm for 30 min	n.d.	n.d.	1 m NaPF_6_ in EC/DEC	1st cycle – 116.5 mAh g^−1^ 500th cycle – ≈70 mAh g^−1^	Between 60% and 75% over 500 cycles
Luna‐Lama et al. 2019^[^ ^44^ ^]^	Ball milled at 350 rpm for 10 min and then heated to 800 °C for 2 h under N_2_ atmosphere	No activation step	<10 m^2^ g^−1^	n.d.	1 m LiPF_6_ in EC/DEC	1st cycle – 764 mAh g^−1^ 2nd cycle – 359 mAh g^−1^ *R*_c_ – ≈ 285 ± 5 mAh g^−1^ (0.1 A g^−1^)	Slights drops observed for first 10 cycles, remains steady thereafter
Djuandhi et al. 2021^[^ ^45^ ^]^	Dried at 9 bar and 95 °C, which was followed by pyrolysis at 900 °C for 15 min under N_2_ flow (1 L min^−1^)	No activation step	1.061 m^2^ g^−1^	0.00634 cm^3^ g^−1^ Avg. Ø 9.7 nm	0.38 m LiTFSI and 0.32 m LiNO_3_ in a 1:1 v/v mix of DOL and DME	489 mAh g^−1^ at 0.1 C	≈70% after 100 cycles (plateau 340 mA h)
Venugopal et al.^[^ ^46^ ^]^	Dried at 100 °C overnight and then pyrolyzed in a tube furnace at 700 °C for 3 h	One step synthesis: coffee waste was mixed with KOH (2:1)	n.d.	n.d.	1 m LiPF_6_ in EC and DEC	1st: 1838 mAh g^−1^ 2nd: 1742 mAh g^−1^	94.8% coulombic efficiency
Abbas et al. 2021^[^ ^47^ ^]^	Sun dried and then oven dried at 60 °C; Pyrolysis at 850 °C for 2–4 h under N_2_ gas flow (150 mL min^−1^)	No activation step	2 h: 72.2 m^2^ g^−1^ 3 h: 72.3 m^2^ g^−1^ 4 h: 36.4 m^2^ g^−1^	2 h: 0.037 cm^3^ g^−1^ 3 h: 0.036 cm^3^ g^−1^ 4 h: 0.019 cm^3^ g^−1^	1.6 m V^3.5+^ in 4 m H_2_SO_4_	n.d.	Coulombic: 98% Energy: 93% Voltage: 94%
Xie et al. 2021^[^ ^48^ ^]^	Dried at 120 °C and then pyrolyzed in tube furnace at 750 °C for 2 h under an Ar atmosphere	Fe(NO_3_)_3_·9H_2_O and CaCO_3_ were added and ball milled for 5 h at 600 rpm prior to carbonization.	No additives: 1.0 m^2^ g^−1^ 3 g of Fe and 20 g CaCO_3_: 531.3 m^2^ g^−1^	No additives: 0.020 cm^3^ g^−1^ 3 g of Fe and 20 g CaCO_3_: 1.547 cm^3^ g^−1^	1 m LiPF_6_ in EC and DEC	Initial discharge of 1523 mAh g^−1^	45% for second discharge and upward of 90% from the 2nd to 50th cycle
Liu et al. 2022^[^ ^49^ ^]^	WCG were mixed with Si@SiO*_x_* and the sintered at 900 °C for 1 h under an Ar gas flow	No activation step	n.d.	n.d.	1 m LiPF_6_ in EC, EMC and 2%FEC	1125 mAh g^−1^ at 100 mA g	80% after 100 cycles at 1 mA g^−1^

After pyrolysis, samples are typically activated using different methods, such as chemical activation with various types of reagents. This is to aid the increase of samples’ porosity and active surface area, which are another two commonly explored properties of WCG derived carbon electrodes manufactured for electrochemical applications. Studies have shown that WCG derived carbon would feature minimal surface area and micro‐porosity without any activation.^[^ ^37^, ^45^ ^]^ It is generally agreed that the specific capacitance (*C*_F_) and charge/discharge ability of the electrode increases with porosity and surface area,^[^ ^4^ ^]^ as increased porosity allows for more charge interactions and pathways to occur.^[^ ^12^ ^]^ More specifically, micropores (diameters < 2 nm) contribute to high surface area, which plays an essential role for charging the electrical double layer capacitance and determines the value of capacitance; on the other hand, mesopores (diameters 2–50 nm) allow fast mass‐transport and the free movement of charged ions providing high power density.^[^ ^104^ ^]^ Recent studies suggested that the coexistence of the microporous and mesoporous structures in carbon materials could have a synergistic effect on their pseudocapacitance of the double layer.^[^ ^105^, ^106^ ^]^ Shi first published a simplified model to estimate the specific capacitive contributions from meso‐ and micropores.^[^ ^107^ ^]^ Rufford et al. later modified this model and discovered that mesoporous structure favors the rapid charge–discharge rate but microporous structure plays a bigger part in the slow charge–discharge process and storing energy.^[^ ^108^ ^]^

#### 4.1.1 Chemical Activation Agents

Rufford et al. first studied the effect of different activation reagents on morphological characteristics and electrochemical properties of carbon electrodes prepared from WCG.^[^ ^3^ ^]^ Coffee grounds were impregnated with FeCl_3_, ZnCl_2_ and MgCl_2_ and then treated at 900 °C. Resultant carbon materials from ZnCl_2_ and FeCl_3_ activation was found to have higher surface areas (977 and 846 m^2^ g^−1^, respectively) than that from MgCl_2_ activation (123 m^2^ g^−1^). As a result, improved specific electrochemical double‐layer capacitances of 57 F g^−1^ was reported in the system with FeCl_3_ treated carbon electrodes.

**Figure 5:**
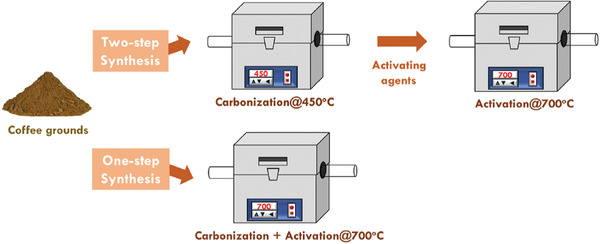
Illustration for preparing activated carbon using the traditional two‐step synthesis and the novel one‐step synthesis for carbonization and activation of waste biomass such as coffee grounds. Reproduced with permission.^[^ ^18^ ^]^ Copyright 2019, Elsevier.

**Figure 6:**
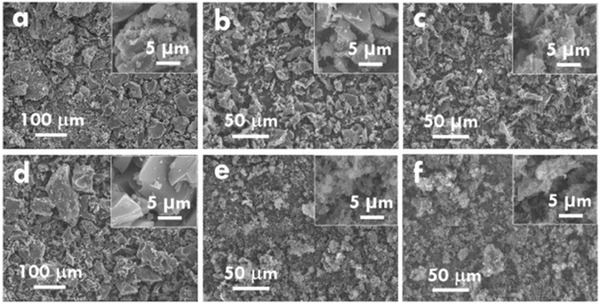
The SEM images for the activated carbons activated using a) H_3_PO_4_, b) HCl, c) FeCl_3_, d) ZnCl_2_, e) NaOH, and f) KOH. Reproduced with permission.^[^ ^18^ ^]^ Copyright 2019, Elsevier.

A later study by Chiu and Lin^[^ ^18^ ^]^ tried to expand the range of activating agents, which encompassed the whole pH range from alkaline and acidic. As a result, a total of six different activating agents (i.e., KOH, NaOH, HCl, H_3_PO_4_, ZnCl_2_, and FeCl_3_) were selected and their effects on activated carbon produced from WCG were investigated using a facile one‐step synthesis method. A mixture of dried WCG and different activating agents (weight ratio 1:2) was placed in a tube furnace and pyrolyzed at 700 °C for 2 h in a N_2_ rich atmosphere. Compared with traditional two‐step pyrolysis (as illustrated in **Figure** **5**), the energy consumption per sample of this modified approach would potentially be reduced. Morphological characteristics and electrochemical properties varied vastly across WCG samples pyrolyzed and activated by the six different activating agents. In **Figure** **6**, SEM images of samples activated by H_3_PO_4_ (a) and ZnCl_2_ (d) appears to have larger grains than those activated by the rest of agents. In terms of surface area (*S*_BET_), KOH activated was the best performing sample, which recorded 1250 m^2^ g^−1^ and was followed by ZnCl_2_ NaOH, H_3_PO_4_, FeCl_3_, and HCl at 1242, 669, 75, 41, and 9 m^2^ g^−1^, respectively. Compared with alkaline activating agents, the more acidic types (HCl, H_3_PO_4_, and FeCl_3_) was proved to be ineffective in creating large specific surface area and enabling pore formation, further evidenced by their lower total pore volume (*V*_total_) recorded at 0.005, 0.041, and 0.035 cm^3^ g^−1^, respectively. The estimated series resistance (*R*_s_) for the electrode was used to indicate the electrical conductivity,^[^ ^109^ ^]^ of which the values were measured for KOH, NaOH, HCl, H_3_PO_4_, ZnCl_2_, and FeCl_3_ to be 3.17, 3.38, 2.12, 2.20, 3.41, 2.66 ohm, respectively. This was found to be roughly in the same trend as their *I*_D_/*I*_G_ ratios (i.e., 2.44, 2.67, 3.11, 0.8, 2.65, and 1.66). Acidic activating agents (HCl, H_3_PO_4_, and FeCl_3_) appeared to produce slightly higher conductivity in the resultant carbon samples. Cyclic voltammetry (CV) and galvanostatic charge‐discharge (GCD) curves were analyzed to calculate the *C*_F_ of each activating agent. *C*_F_ values of 105.3, 69.5, 0.6, 51.0, 72.9, and 41.3 F g^−1^ were recorded for KOH, NaOH, HCl, H_3_PO_4_, ZnCl_2_, and FeCl_3_, respectively. The best *C*_F_ achieved by KOH activated samples was shown to be five times of that of commercial activated carbon electrode. Symmetric capacitor fabricated using the best WCG derived samples displayed the maximum energy density of 6.94 Wh kg^−1^ at the power density of 350 W kg^−1^ along with excellent cycling stability after 8000 cycles. In the study, the highest *C*_F_ value for the KOH‐activated WCG electrodes was attributed to their largest specific surface area, the suitable pore volume for ions adsorption and diffusion, and the less hydrophobic functional groups (better contact between electrode surface and electrolytes); on the other hand, moderate *I*_D_/*I*_G_ ratio (≈2.5) was essential in achieving the delicate balance of sufficient active sites and electrical conductivity simultaneously. In addition, the observed shifts of the D‐band and G‐band peaks to the higher values for the KOH‐activated carbon could imply a shorter bond length, which also favors higher charge transfer and thus the energy storage ability.

So far, most reported activating agent for WCG pyrolytic conversion was KOH, and various chemical activation reactions using the KOH have been proposed in Equations (1)–(6) as follows.^[^ ^110^ ^]^ The high basic property and small size of the potassium ions (intercalating with carbon lattices) contribute to the superior activation ability of KOH toward efficient conversion of WCG to activated carbon materials^[^ ^111^ ^]^

(1) $$\begin{array}{c} 4\text{KOH}+\left(-{\text{CH}}_{2}\right)\to K_{2}{\text{CO}}_{3}+K_{2}O+H_{2} \end{array}$$

(2) $$\begin{array}{c} 6\text{KOH}+2C\to 2K_{2}{\text{CO}}_{3}+2K+3H_{2} \end{array}$$

(3) $$\begin{array}{c} K_{2}{\text{CO}}_{3}\to {\text{CO}}_{2}+K_{2}O \end{array}$$

(4) $$\begin{array}{c} 4\text{KOH}+C\to 4K+{\text{CO}}_{2}+2H_{2}O \end{array}$$

(5) $$\begin{array}{c} K_{2}O+C\to 2K+\text{CO} \end{array}$$

(6) $$\begin{array}{c} K_{2}{\text{CO}}_{3}+2C\to 2K+3\text{CO} \end{array}$$

**Figure 7:**
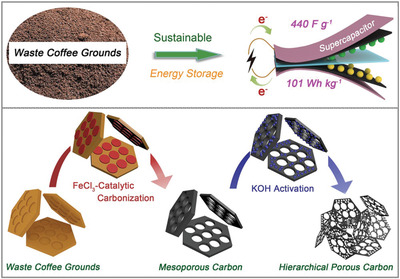
Synthesis process of hierarchical porous carbon from waste coffee grounds and its supercapacitor application. Reproduced with permission.^[^ ^21^ ^]^ Copyright 2020, Springer Nature.

**Figure 8:**
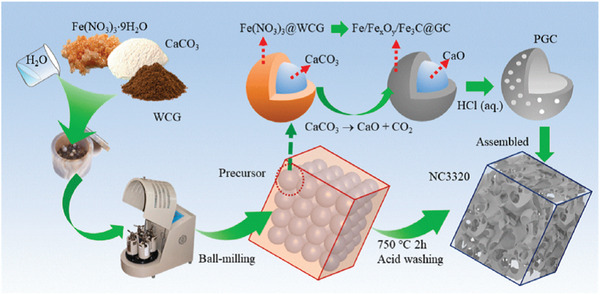
Preparation scheme of the nitrogen‐enriched graphene‐like nanosheets constructed 3D porous carbon framework. Reproduced with permission.^[^ ^48^ ^]^ Copyright 2021, Elsevier.

Multistep chemical activation of WCG with more than one agent has also been reported. In the study by Liu et al., a mixture of WCG and FeCl_3_ with weight ratio of 5:1 was first sonicated and pyrolyzed at 700 °C (10 °C min^−1^) under argon for 2 h, which was followed by a second stage of pyrolysis with different amount of KOH heated at 800 °C (5 °C min^−1^) for 2 h under argon (see **Figure** **7**).^[^ ^21^ ^]^ The FeCl_3_ was shown to produce mainly microporosity and the following KOH activation introduced a large amount of micropores, which together created the hierarchical porous structure. A high carbon yield of 42.5 wt% from WCG was highlighted, as adsorbed iron‐containing particles in the well‐defined mesoporous carbon structure could play a catalyst role during the high temperature carbonization process of biomass/polymer.^[^ ^112^ ^]^ As illustrated in **Figure** **8**, Xie et al. demonstrated the combined use of Fe(NO_3_)_3_ as graphitization catalyst and CaCO_3_ (plus its CO_2_ by‐product) as pore forming agent during a pyrolysis of WCG (750 °C, Ar, 2 h), which led to a 3D porous carbon architecture consisting of crosslinked, wrinkled carbon nanosheets (around five layers).^[^ ^48^ ^]^ Research has shown that corresponding derivatives (Fe/Fe*_x_* O*_y_*/Fe_3_C, etc.) of iron‐containing precursors could easily grow into carbonaceous structure with high temperature.^[^ ^113^ ^]^

#### 4.1.2 Chemical Activation Temperature

**Figure 9:**
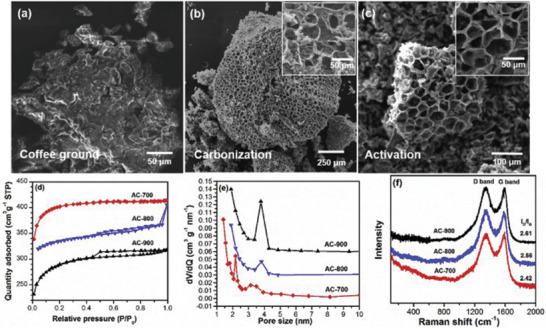
SEM images of waste coffee grounds (WCG) a) as received, b) after carbonization, and c) after activation (AC‐700); d) N_2_ adsorption isotherms, e) pore size distribution, and f) Raman spectra of AC‐700, AC‐800, and AC‐900. Reproduced with permission.^[^ ^41^ ^]^ Copyright 2019, Elsevier.

Activation temperature could have effect on the performance of WCG derived electrodes when using different activating agents. Zhao et al. examined the effect of activation temperature when using KOH as the activating agent (WCG:KOH mass ratio of 2:1) following pyrolytic carbonization (700 °C, 10 °C min^−1^ under N_2_ atmosphere).^[^ ^41^ ^]^ The mixture was then annealed at 700, 800, and 900 °C for 2 h in a N_2_ rich atmosphere. Their study found that the activation at 700 °C yielded the largest specific surface area (1355 m^2^ g^−1^) and greatest volume of micropores, while higher temperatures reduced the total specific surface area as the pores may have collapsed in the heat. Further SEM images (**Figure** **9**) showed that the carbonization formed a honeycomb‐like structure with small pores across the sample. Upon the activation, the same honeycomb shape was maintained, while the pore size appeared to have increased drastically. This contrasts with the raw WCG that has a wrinkled surface and a nonporous structure, as evidenced in SEM analysis.^[^ ^114^ ^]^ It was also observed that the intensity *D* (*I*_D_, ≈1300 cm^−1^) and *G* (*I*_G_, ≈1600 cm^−1^) bands in Raman spectroscopy were slightly different across the different activation temperatures used within the study. Results for the *I*_D_/*I*_G_ ratio were 2.42, 2.56, and 2.61 for 700, 800, and 900 °C, respectively. The lowest *I*_D_/*I*_G_ ratio of 700 °C sample indicated a slight increase in the graphitization and thus better conductivity of the materials as the G band is associated with the in‐plane vibrations of ordered sp^2^ carbons, whereas the D band is the outer plane vibrations in structural defects and disordered sp^3^ carbons. WCG derived carbon was then utilized as a selenium (Se) cathode host in Li–Se batteries by a melting diffusion method. At a scanning rate of 0.1 mV s^−1^, the reversible specific capacity of Se/AC‐700 was 655 mAh g^−1^, which is close to the theoretical value of Se (675 mAh g^−1^). Based on Nyquist plot simulation results, electron transfer and Li‐ion diffusion coefficient of Se/AC‐700 was calculated to be the highest, which led to the excellent performance as the composite cathode. The cycling stability was also assessed in which Se/AC‐700 recorded at 631 mAh g^−1^ after 100 cycles, with insignificant capacity decay of 0.04% probably due to Se confined tightly in the microporous carbon. Some of these findings are consistent with the previous study by Wang et al., which also reported that 700 °C for KOH activation of WCG was the optimal temperature to produce the highest specific surface area (1622.77 m^2^ g^−1^) and capacitance (175 F g^−1^ at 1 A g^−1^).^[^ ^13^ ^]^

**Figure 10:**
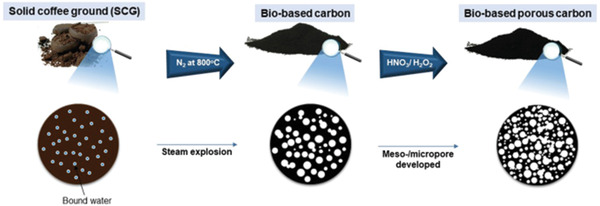
A schematic illustration of the synthesis of hierarchical porous carbon from raw spent coffee ground. Reproduced with permission.^[^ ^40^ ^]^ Copyright 2020, Springer Nature.

A study by Kim et al.^[^ ^40^ ^]^ investigated the effect of activation time on WCG derived carbon using 2 m HNO_3_ and 3% w/w H_2_O_2_ after their pyrolysis (800 °C under N_2_ atmosphere). The acid mixture of HNO_3_ and H_2_O_2_ could introduce higher porosity and surface area because it would penetrate the carbon layers and break them up by oxidation.^[^ ^115^ ^]^ Wet WCG samples were either left untreated (at 61% w/w bound water) and denoted as W‐SPC, while the WCG samples dried in a convection oven at 110 °C for 24 h were denoted as D‐SPC. The activation temperature was altered to investigate its effect at 60, 80, and 100 °C for 24 h. D‐SPC samples exhibited specific surface areas of 31.69, 256.34, 636.62, and 529.30 m^2^ g^−1^ for no activation, 60, 80, and 100 °C, respectively; W‐SPC samples exhibited specific surface areas of 510.85, 860.68, 1037.52, and 716.83 m^2^ g^−1^ for no activation, 60, 80, and 100 °C, respectively. Generally, the higher activation temperature accelerates the reaction, enabling pore formation and enlargement in the carbon structure.^[^ ^116^, ^117^ ^]^ This is consistent with the reported results and showed that the activation at 80 °C was the most effective at increasing the specific surface area and mesoporosity of the WCG. However, the higher activation temperature (>80 °C) induced excessive oxidative degradation on the surface of WCG, causing mesoporous structures to collapse and the total specific surface area to diminish. Compared with the highest specific surface area of D‐SPC samples at 80 °C, W‐SPC activated at the same temperature showed even higher specific surface area. This is potentially due to the formation of larger pores when the bound water vapor exploded within the W‐SPC structure during the pyrolysis. As illustrated in **Figure** **10**, these pores could have been further opened and enlarged from the chemical activation resulting in additional micro‐ and meso‐porosity. Sulfur impregnated W‐SPC‐80 (weight ratio of 1:4) were then tested as electrodes in Li–S battery in the study, where an excellent initial discharge capacity of 1458 mAh g^−1^ was recorded and very close to the theoretical capacity of sulfur (1675 mAh g^−1^). This was also corroborated by the nearly vertical line in the lower frequency region of Nyquist plot, indicating enhanced diffusion of lithium ions into the hierarchical structure of W‐SPC‐80.^[^ ^118^ ^]^ Upon testing the cycling stability, this value rapidly declined for the first 50 cycles and then stabilized with an average decrease of 0.3% per cycle. The rapid decline of discharging capacity after the first cycle was ascribed to the desorption of some of the sulfur and converted into Li_2_S at the edge of the carbon and their subsequent electrochemical isolation;^[^ ^119^ ^]^ Much slower decrease from the second cycle was due to Li_2_S dendrite formed on the lithium electrode surface during the charging process, leading to a decrease in the total amount of sulfur participating in the charge/discharge process and the irreversible capacity reduction.^[^ ^120^ ^]^ A final charge/discharge capacity of 474 mAh g^−1^ at current density of 0.2 mA cm^−2^ was maintained without further deterioration after 400 cycles. The similar phenomena of stabilized capacity after multicycling were also observed in other studies by Djuandhi et al.^[^ ^45^ ^]^ and Gao et al..^[^ ^37^ ^]^ They assigned the cause to the growth of solids electrolyte interphase (SEI) layer, which could slow down formation of dendrite to some extent and thus improve cell cycling stability.

#### 4.1.3 CO_2_ Activation

Tashima et al. reported a study using the low‐cost CO_2_ as a form of physical activation for pyrolyzed WCG at 600 °C for 1 h under N_2_ flow (700 mL min^−1^).^[^ ^7^ ^]^ Every sample was then subjected to activation at 1000 °C at a constant heating rate of 11 °C min^−1^ with a constant CO_2_ gas flow (700 mL min^−1^). They then investigated the impacts that the activation time could have on the surface area and porosity of pyrolyzed WCG. Six samples were carbonized at a heating rate of 10 °C min^−1^ and then activated for 1–6 h at 1 h increment were denoted as HC600A1‐6; Three samples were carbonized at a heating rate of 5 °C min^−1^ and then activated for 1–3 h at 1 h increment were denoted as HC300A1‐3. The results obtained from this study highlighted that the highest specific surface area was obtained after 2 h of activation, with HC600A2 and HC300A2 having a specific surface area of 1596 and 1867 m^2^ g^−1^, respectively. Excessive activation demonstrated a detrimental effect on the specific surface area. After 2 h of activation, there was a rapid decrease in the specific surface area of the materials, e.g., longest activated time would reduce the specific surface area down to 258 m^2^ g^−1^ for HC600A6. The same trend for peak micropore volume (i.e., 0.769 cm^3^ g^−1^ and 0.958 cm^3^ g^−1^) were observed after 2 h of activation for HC600A2 and HC300A2, respectively, whereas the lower heating rate (i.e., 5 °C min^−1^) in the pyrolysis step proved to increase the pore volume further. Longer activation time was also found to reduce the oxygen‐containing functional group (e.g., the phenolic hydroxyl groups and the carboxyl group), which was turned into gas by the reaction of carbon and oxygen. This is potentially another drawback because the carboxyl group has been reported to give rise to the values of specific capacitances in aqueous electrolytes.^[^ ^121^ ^]^

Electrochemical performances of WCG derived electrode materials were assessed as electric double layer capacitor (EDLC), which normally have better lifecycle than a typical battery with excellent discharge characteristics. The CV analysis was conducted at a scan rate of 10 mV s^−1^, where the integrated area of quasi‐rectangular cyclic voltammogram for each sample mainly correlated with its BET surface area, as the higher specific surface areas allowed for more charge transfer and therefore a better calculated areal capacitance. Therefore, HC300A2 with the highest specific surface area and greatest porosity was marked as the best performing sample with a recorded specific capacitance of 103 F g^−1^ in the aqueous electrolyte (1 m H_2_SO_4_) and that of 152 F g^−1^ in the organic electrolyte (0.8 m tetraethylammonium tetrafluoroborate [(C_2_H_5_)_4_NBF_4_] in propylene carbonate). Finally, the cycling stability of HC300A2 sample was tested at the scan rate of 10 mV s^−1^, where its specific capacitance remained in the 130–150 F g^−1^ range showing no significant drops or fading throughout 3000 cycles.

#### 4.1.4 Steam activation

In the study performed by Krikstolaityte et al, they used coffee beans for an electrode material in vanadium‐redox batteries (VRB) and investigated the activation step using steam alone, where water vapor and carbon material normally undergo the steam reforming reaction between 700 and 800 °C, producing CO + H_2_ and yielding the porous structure.^[^ ^36^ ^]^ Dried and pelletized coffee beans was first pyrolyzed at 850 °C (heating rate of 5 °C min^−1^) in a quartz tube for 30 mins under a N_2_ gas flow (250 mL min^−1^). Following the pyrolysis, samples were subjected to steam activation using a 63 vol% steam and 37 vol% N_2_ at a total flow rate of 670 mL min^−1^ at various activation times (1, 2, and 3 h). The impact of activation time on sample's specific surface area and the kinetics of the electrochemical reactions was assessed. Narrow pores are typically formed at first, which would progressively widen with time increasing the specific surface area and diminishing the charge transfer resistance. However, prolonged activation process was also found to increase microporosity/structural disorders in WCG derived carbon, and thus be responsible to cause stagnant ion mass transport and reduction of the overall energy/voltage efficiency.^[^ ^122^ ^]^

#### 4.1.5 Dopant Effect

**Figure 11:**
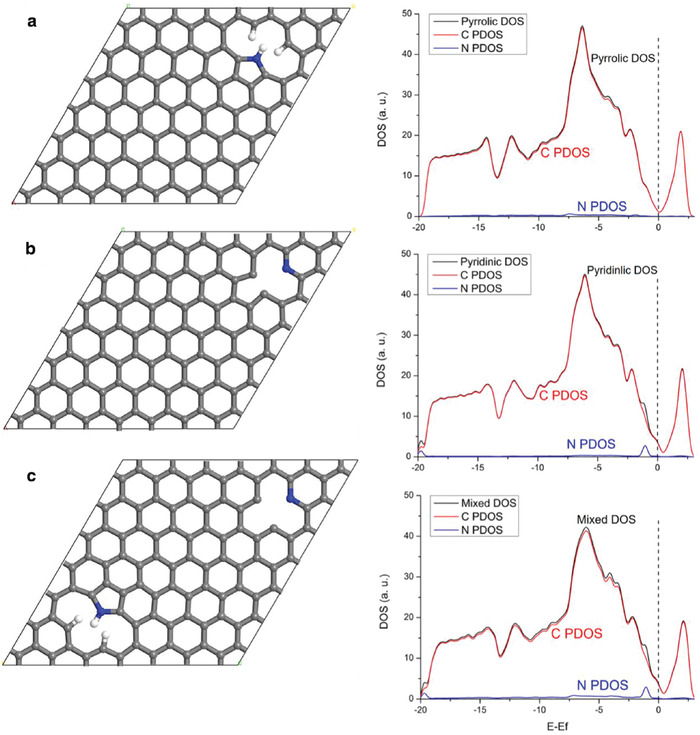
DFT Models used calculations and the corresponding density of state plots for a) pyrrolic, b) pyridinic, and c) mixed. Black curve is total density of states. Reproduced with permission.^[^ ^39^ ^]^ Copyright 2019, Elsevier.

During the pyrolysis under various atmospheres, dopants can be introduced into carbonized WCG matrix. For example, raw WCG contains nitrogen heteroatoms (2.38% w/w) naturally, which would enable a certain degree of self‐doping of nitrogen.^[^ ^40^ ^]^ As a result, the potential effects of different dopants on the performance of WCG derived electrodes have also been investigated in applications, such as Li/Na‐ion battery^[^ ^39^ ^]^ and supercapacitors.^[^ ^13^, ^14^, ^17^ ^]^ So far, nitrogen as a dopant received most of the attention as the C–N bond is weaker than the C–O, H–N, and C–H bonds and thus can be easily engraved on the coffee grounds surface.^[^ ^123^ ^]^ Tsai et al. reported using adamantane‐like structure HMT (C_6_H_12_N_4_) as nitrogen precursor for doping the WCG samples, which thermally decompose into NH_3_ atmosphere with no residue observed after 200 °C^[^ ^39^ ^]^ and reduce the oxygen components in WCG by removing CO, CO_2_, or water molecules during the pyrolysis. High nitrogen content of 6.17% (more in the pyridinic form) was observed in nitrogen doped samples. Compared with pyrrolic nitrogen, the pyridinic N rich carbon sample with relatively lower degree of graphitization could contribute to more electrochemical active sites and efficient interfacial contact between electrode and electrolytes. Their density functional theory (DFT) calculations further indicated that the presence of pyridinic N introduced extra states near the Dirac cone (see **Figure** **11**), which could result in greater conductivity. These findings make pyridinic graphene most suit able for Li storage as they enhance discharge capacity and promote the Li/Na‐ion storage performances.^[^ ^124^ ^]^ Consequently, nitrogen doped WCG electrodes showed a power density of 107.1 mAh g^−1^, which was nearly 2.02 times than that of undoped samples.

Another main advantage of nitrogen doping is the increased mesoporosity within carbon matrix, which could enhance diffusivity by introducing more paths of ion diffusion. Both Choi et al.^[^ ^17^ ^]^ and Wang et al.^[^ ^13^ ^]^ reported that nitrogen introduction in presence of melamine and *N*‐methyl pyrrolidinone (NMP) led to the high specific surface area (1824 and 1622.77 m^2^ g^−1^, respectively) for activated carbon derived from WCG, in which the presence of pyridinic‐N, pyrrolic‐N, and pyridine‐N‐oxide were observed. Both pyridinic‐ and pyrrolic‐N containing functional groups in carbon formation are known to be electrochemically active. On the other hand, the positive charge on pyridine‐N‐oxide would enhance the electron transfer at the carbon/aqueous interface.^[^ ^125^, ^126^ ^]^ As a result, high specific capacitance of 74 and 175 F g^−1^ was reported at a current density of 1 A g^−1^ as supercapacitors in their studies, respectively, together with remarkable cycling stability (10 000 cycles test).

**Figure 12:**
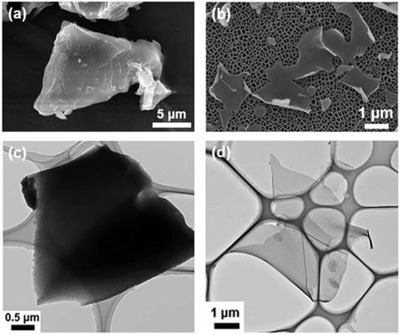
Scanning electron micrographs of a) C‐WCGs) and b) nanoporous carbon nanosheets (NCNSs), and transmission electron micrographs of c) C‐WCGs and d) NCNSs. (C‐WCGs are obtained the same way as the NCNSs but with the absence of an activating agent [KOH]). Reproduced with permission.^[^ ^14^ ^]^ Copyright 2016, Korean Journal Publishing Service.

Park et al. found that the combined presence of several dopants (16.1 at% oxygen, 2.7 at% nitrogen, and 1.6 at% sulfur) could aid the performance of capacitors derived from carbonized WCG.^[^ ^14^ ^]^ High specific surface area (1960.1 m^2^ g^−1^), of which most were micropores (1932.5 m^2^ g^−1^) ≈0.6 nm in size, and specific capacitance (best 438.5 F g^−1^ at 2 mV s^−1^) was obtained with good cycling stability (2000 cycles). Unfortunately, direct comparison with samples containing no dopants was not carried out in their study. Interestingly, high temperature (800 °C) KOH activation was discovered to exfoliate amorphous carbon structures into nanoporous (<100 nm diameter) carbon nanosheets (NCNS) of defective hexagonal carbon structures with high aspect ratios (>100), as shown in **Figure** **12**b,d.

In studying phosphorus containing functional groups and their effects on the carbon surface, particularly the polyphosphates (i.e., P_2_O_7_^4−^, PO_3_^−^, and P_4_O_10_), Huang et al. reported a high specific capacitance of 180 F g^−1^ (at a current density of 1 A g^−1^) and energy density of 15 Wh kg^−1^ in supercapacitors assembled with carbonized WCG via 800 °C H_3_PO_4_ activation.^[^ ^5^ ^]^ A stable energy storage performance was achieved over 1.5 V, which was even above the theoretical potential (1.23 V) for water splitting. This widening of working potential window was ascribed to the reversible electrochemical hydrogen storage in narrow carbon micropores stabilized by the phosphorus functional groups. Based on an unexplained pair of redox peaks at −0.4 V/−0.1 V versus Ag/AgCl in CV voltammograms, they also speculated that these polyphosphates on carbon surface probably participated in certain redox reactions, which could contribute to the improvement of energy storage.

### 4.2 Microwave Assisted Carbonization

**Figure 13:**
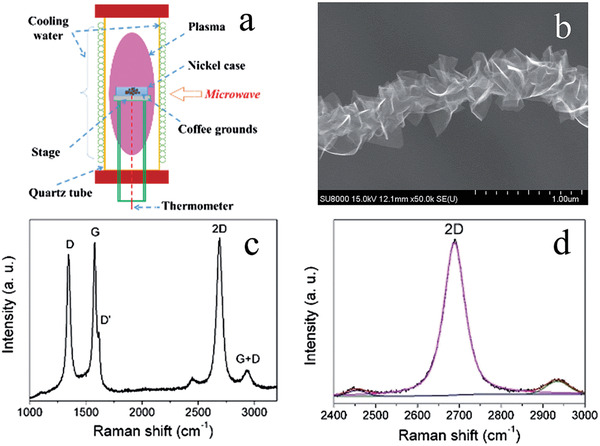
A schematic diagram of the experiment, and the morphology and microstructure of individual GSFs obtained from waste coffee grounds by the MPI technique. a) Setup of the MPI technique. b) SEM image of a GSF. c) Raman spectrum obtained with the laser at 532 nm, and d) 2D peak in the Raman spectrum fitted by a single Lorentzian function. Reproduced with permission.^[^ ^8^ ^]^ Copyright 2015, Royal Society of Chemistry.

**Figure 14:**
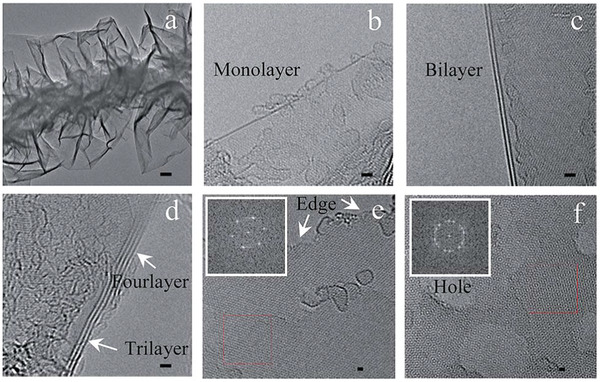
TEM images of an individual GSF. a) A graphitic fiber without a hollow structure consisted of graphene nanosheets with a varying thickness. b) Monolayer graphene. c) Bilayer graphene. d) Tri‐ and four‐layer graphene. e) High‐resolution TEM image from monolayer graphene. Inset: a hexagonal pattern of carbon atoms by fast Fourier transform for monolayer graphene. f) High‐resolution TEM image from few‐layer graphene. Inset: a dotted ring pattern for few layer graphene. Scale bars: a) 50 mm, b–d) 1 nm, and e,f) 0.5 nm. Reproduced with permission.^[^ ^8^ ^]^ Copyright 2015, Royal Society of Chemistry.

In 2015, Wang et al. highlighted a novel alternative carbonization approach, utilizing microwave plasma irradiation (MPI) to generate the various carbonaceous material from WCG.^[^ ^8^ ^]^ Plasma ignition of H_2_–Ar mix (1:1 ratio) was achieved by using a 2.45 GHz microwave at 900 W, the WCG were loaded into a quartz tube in a nickel foil case and then bombarded with the plasma mixture for 15 mins as illustrated in **Figure** **13**a. Under these conditions, WCG generated carbon atoms, which could then be deposited as formation of the carbon layer, leading to spherical particles with energetic dangling bonds. As temperature and pressure increased, carbon nanotubes were produced with the aid of nickel particles. As a result, the use of MPI allowed for long wavy graphene‐sheet fibers (GSF) of various types (carbon nanotubes with different layer graphene sheets grown on their sidewalls) and sizing (a diameter within the range of 10–200 nm) to be produced from the WCG (see Figure 13b). Along with the presence of D and G bands, Raman spectroscopy analysis showed characteristic peaks to that of monolayer graphene, where the 2D peak (≈2700 cm^−1^) with the single bandwidth of ≈60 cm^−1^ was observed (see Figure 13c,d). High resolution TEM also confirmed the stacking configuration, which consisted of mono‐, bi, and trilayer graphene, with a spacing between the layers of 0.345–0.352 nm (see **Figure** **14**).

To illustrate the electrochemical performance of WCG derived GSF, CV analysis was conducted across the potential of 0.0–0.5 V in 1 m KCl solution, where typical rectangular shapes of *I*–*V* curves were observed for scan rates up to 0.1 V s^−1^ indicating a good capacitive behavior. Specific capacitance was calculated to be 223.93, 71.05, and 1293.33 µF g^−1^ at a scan rate of 0.1 V s^−1^ for a bare Pt, glassy carbon (GC), and modified WCG derived graphene electrode, respectively. This huge improvement in terms of the capacitance was attributed to the increased specific surface areas and unique morphology of the resultant GSF. This has demonstrated that the obtained WSG derived GSF is highly suitable for electrochemical conversion and energy storage applications, especially as capacitors.

## 5 Current Challenges and Future Research Directions

As one of the largest contributors to food waste, WCG has created adverse environmental burden due to their decomposition. Recycling WCG into other materials for use is a key step into reducing global waste in the beverage industry and play a significant role in societal transitioning to food circular economy. WCG contains mainly lignocellulose, of which the carbonization has been shown to be achievable via relatively rapid pyrolysis. In the recent decade, there are growing interests in converting abundant WCG into value‐added carbon electrode materials. These WCG‐derived electrodes have demonstrated their potential in various electrochemical applications owing to their low cost and high performance, such as batteries, supercapacitors, and sensors. In this context, the recent progress on using WCG as a renewable carbon source to produce electrode materials has been reviewed, where some of key challenges and potential research directions are highlighted as follows:

1. The impregnation ratios of activating agents to WCG could affect surface area and electrochemical performance significantly, as pore size distribution can be controlled by adjusting the impregnation ratio. For example, ZnCl_2_ to WCG weight ratio of 2 was reported to be optimal consistently in terms of producing larger pores, especially in range of >2 nm.^[^ ^2^, ^107^ ^]^ The similar optimal ratio and results were observed when using the H_3_PO_4_,^[^ ^5^ ^]^ while a ratio of 4 was optimal for KOH in terms of specific capacitance.^[^ ^21^ ^]^ However, studies of this nature have not been systematically carried out for each of activating agents.
2. In summary, electrochemical performance (e.g., specific capacitance and energy density) of the WCG derived electrodes are more likely to depend on the combined effect of several factors: 1) the amount of the hydrophobic and hydrophilic functional groups (i.e., active sites), 2) the *I*_D_/*I*_G_ ratio, as well as 3) the specific surface area and pore structure. However, it should be noted that correlating these factors and the properties of WCG derived carbon electrodes is intrinsically challenging, particularly in an attempt to precisely determine the factors or their combinations that are responsible for observed improvements in electrochemical performance. For example, finding the ratio of macro‐, meso‐, and microporosity and correlating it to the ideal electrochemical performance in a disordered carbon structure is in itself extremely complex.
3. It might not be able to compare some of electrochemical performance directly in the literature. For example, specific capacitance measurement depends on the electrolytes used (e.g., types and concentrations), which are widely different across reported studies.
4. While pyrolysis is still most commonly reported method to convert WCG, there are other techniques to convert WCG into electrode materials, such as plasma jet assisted carbonization^[^ ^127^ ^]^ and photonic annealing under inert atmosphere.^[^ ^128^ ^]^ This can be applied and optimized to produce WCG derived electrode materials.
5. The “zero‐waste” approach in reusing WCG as electrode materials has been incorporated into different scientific research avenues and a variety of applications (mainly energy storage devices). Application of WCG‐derived carbon as electrochemical sensors can further boost this approach in food circular economy, and yet only two studies have been reported so far in this promising area.^[^ ^91^, ^92^ ^]^ Moreover, in line with the recent development of various high‐volume printing techniques, molecular imprinting, microfluidics and other relevant nanofabrication techniques, the mass production of cheap deposable electrochemical sensors from WCG has shown a great potential.^[^ ^127^, ^129^, ^130^, ^131^, ^132^, ^133^ ^]^

## Conflict of Interest

The authors declare no conflict of interest.
